# Down syndrome and the molecular pathogenesis resulting from trisomy of human chromosome 21

**DOI:** 10.1016/S1674-8301(10)60016-4

**Published:** 2010-03

**Authors:** Aarti Ruparelia, Frances Wiseman, Olivia Sheppard, Victor L.J. Tybulewicz, Elizabeth M.C. Fisher

**Affiliations:** a Department of Neurodegenerative Disease, UCL Institute of Neurology, Queen Square, London, UK; b MRC National Institute for Medical Research, The Ridgeway, London, UK

## Abstract

Chromosome copy number aberrations, anueploidies, are common in the human population but generally lethal. However, trisomy of human chromosome 21 is compatible with life and people born with this form of aneuploidy manifest the features of Down syndrome, named after Langdon Down who was a 19^th^ century British physician who first described a group of people with this disorder. Down syndrome includes learning and memory deficits in all cases, as well as many other features which vary in penetrance and expressivity in different people. While Down syndrome clearly has a genetic cause - the extra dose of genes on chromosome 21 - we do not know which genes are important for which aspects of the syndrome, which biochemical pathways are disrupted, or, generally how design therapies to ameliorate the effects of these disruptions. Recently, with new insights gained from studying mouse models of Down syndrome, specific genes and pathways are being shown to be involved in the pathogenesis of the disorder. This is opening the way for exciting new studies of potential therapeutics for aspects of Down syndrome, particularly the learning and memory deficits.

## INTRODUCTION

Down Syndrome (DS) is the consequence of trisomy of human chromosome 21 (Hsa21) and is the most common genetic form of intellectual disability, occurring in approximately 1 in 700 live births[1]. DS is characterised by invariant features that are common to all affected individuals, including mild-to-moderate learning disabilities, craniofacial abnormalities and hypotonia[2],[3]. In addition, at least 80 other variable phenotypes that affect only a proportion of DS individuals have been described, such as an early-onset of Alzheimer's disease, atrioventricular septal heart defects, acute megakaryoblastic leukemia and a decrease in the incidence of some solid tumours[4]–[7]. Significant advances in medical treatment and social care have increased the average life span of people with DS to greater than 60 years[8].

The additional copy of Hsa21 results in elevated expression of many of the genes encoded on this chromosome, with varying expression levels in different tissues[9]–[11]. The increased dosage of Hsa21 genes, and the dosage imbalance between Hsa21 and non-Hsa21 genes has been proposed to cause the plethora of phenotypic alterations that characterize DS. The gene-rich distal part of Hsa21, identified as the ‘Down syndrome critical region’ (DSCR), was initially proposed to be sufficient to cause most of these DS phenotypes[12]–[14]. However, accumulating evidence points against a single DSCR[14],[15]. Current data suggest that a number of ‘susceptibility regions’ located on Hsa21, which are modified by other loci on Hsa21 and elsewhere in the genome, increase the risk of developing specific DS associated phenotypes[14],[15].

**Fig. 1:**
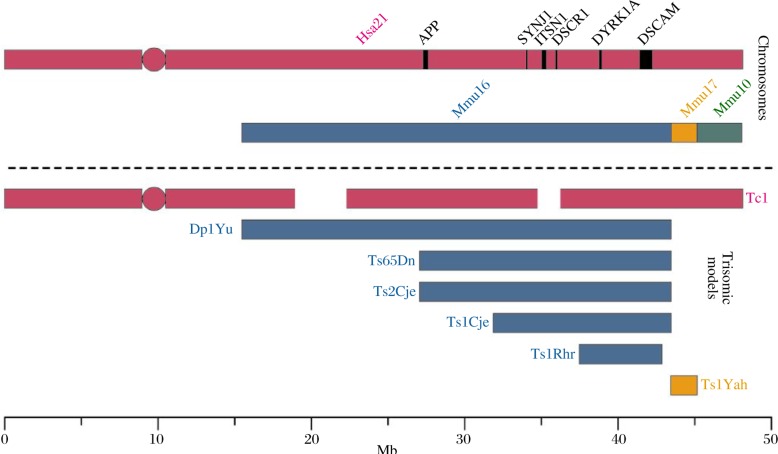
Mouse models of Hsa21 trisomy. Hsa21 (purple) is syntenic with regions of mouse chromosomes 16 (Mmu16, blue), 17 (Mmu17, orange) and 10 (Mmu10, green). The positions of some Hsa21 genes implicated in the pathogenesis of DS and mentioned in this text are shown on the human chromosome. The transchromosomic Tc1 model carries a freely segregating copy of Hsa21 and is trisomic for the majority of genes on Hsa21[33]. Several mouse models are syntenic with a proportion of genes on Hsa21 and are segmentally trisomic for regions of Mmu16, such as the Dp1Yu[18], Ts65Dn[25], Ts2Cje[23], Ts1Cje[24], and Ts1Rhr[19] models. The Ts1Yah mouse model[22] is syntenic to Mmu17 and is trisomic for the sub-telomeric region of Hsa21.

Mouse models of DS are instrumental in identifying which genes contribute to DS phenotypes, and unraveling the mechanisms by which these phenotypes arise[16]–[24]. Hsa21 is syntenic to three regions of the mouse genome. Most of the genes on Hsa21 have homologous genes on mouse chromosome 16 (Mmu16), but two smaller gene rich regions have synteny on Mmu10 and Mmu17 (***Fig. 1***). The majority of mouse models used for DS research are either trisomic for large regions of Mmu16, 10, 17 or are transgenic animals used to investigate overexpression of a single gene[16]–[32]. The Tc1 mouse model, with which we mainly work, carries a freely segregating almost complete copy of Hsa21, in addition to a normal complement of the mouse chromosome[33].

In this review, we highlight recent developments in understanding how overexpression of Hsa21 genes leads to many of the features of DS. We focus on key areas including brain, heart and cancer, as these are currently the most developed in our understanding of the molecular pathogenesis of DS.

## RECENT BREAKTHROUGHS IN OUR UNDERSTANDING OF PHENOTYPES ARISING FROM TRISOMY HSA21

### Learning and Memory

People with DS have learning and memory problems and exhibit differences in brain structure compared to the euploid population[34]–[39]. Mouse models of DS recapitulate these neuroanatomical changes and behavioural deficits, and thus can be used to further our understanding of learning and memory in people with DS[25]. The Ts65Dn mouse model of DS is trisomic for approximately 136 genes on Mmu16 that have homologues on Hsa21[25] (***Fig. 1***). These mice have learning and memory phenotypes and it has been proposed that excess inhibition of synaptic transmission may contribute to their deficits[25],[40]. Recent papers have shown that the structure of receptors and their abundance at inhibitory synapses is altered in the hippocampus of Ts65Dn mice, which provides insight into the neurological changes that may underlie their DS-associated memory problems[41],[42]. In addition, impaired synaptic plasticity was recently demonstrated in Ts65Dn striatal cholinergic interneurons[43], highlighting a potentially novel and important role for the interstriatal cholinergic system in the pathophysiology of DS-associated motor and cognitive defects. The Tc1 mouse model of DS, which is trisomic for approximately 80% Hsa21 genes, has short-term but not long-term deficits in hippocampal-dependent learning and abnormalities in long-term potentiation (LTP), which is proposed to be a physiological correlate of learning[33],[44]. Interestingly, although Tc1 mice display major deficits in cerebellum-dependent learning tasks, no abnormalities in synaptic function or in cerebellar long-term depression can be detected in this model[45].

In the Ts1Rhr mouse model (***Fig. 1***), trisomy of 33 Mmu16 genes that are syntenic to the DSCR and include *dual-specificity tyrosine-* (*Y*)*-phosphorylation-regulated kinase 1A* (*Dyrk1A*), *potassium inwardly-rectifying channel, subfamily J, member 6 Gene* (*Girk2*) and *single-minded homologue 2* (*Sim2*), cause alterations in dendritic spine morphology and deficits in some behavioural tests[46] (***Table 1***). Trisomy of these genes is necessary but not sufficient to elicit Morris water maze learning deficits in mouse DS models[13]. These data indicate that interactions of Hsa21 trisomic genes may contribute to DS-associated learning and memory problems. Trisomy of 12 genes (*Abcg1-U2af1*) found on the Hsa21 sub-telomeric region in Ts1Yah mice (***Fig. 1***), produced cognitive defects in working and short-term recognition memory, but an enhancement of hippocampal-dependent spatial learning[22]. This study is pivotal in showing that variation in copy number is not always deleterious.

The over-expression of a number of Hsa21 genes has been implicated in learning and memory deficits in single gene transgenic mouse models, suggesting that trisomy of these genes may contribute to learning disability in DS individuals. These genes include *DYRK1A*, *synaptojanin 1*(*SYNJ1*) and *SIM2* [26],[28]–[32],[47],[48]. Recent evidence has emerged for a possible role in brain function of *dopey family member 2* (*DOPEY2*)[49] and *Down syndrome cell adhesion molecule* (*DSCAM*)[50], two Hsa21 genes known to be involved in learning and memory.

**Table 1:** Chromosome 21 genes implicated in the pathogenesis of DS phenotypes

DS Phenotype	Implicated Hsa21 Genes	References
Learning and Memory	*DOPEY2*	Rachidi et al., 2009[49]
	*DSCAM*	Yu et al., 2009[50]
	*DYRK1A*	Altafaj et al., 2001[30]
	*SIM2*	Meng et al., 2006[48]
	*SYNJ1*	Voronov et al., 2008[29]
Neurodevelopment	*PREP1*	Micali et al., 2010[59]
	*TTC3*	Suizu et al., 2009[60]
Alzheimer's Disease	*APP*	Rovelet-Lecrux et al., 2006[88];
		Sleegars et al., 2006[89];
		Cabrejo et al., 2006[90];
		Salehi et al., 2006[92];
		Cataldo et al., 2003[97];
		Jiang et al., 2009[100]
	*DYRK1A*	Ryoo et al., 2007[102];
		Ryoo et al., 2008[103];
		Liu et al., 2008[105];
	*ITSN1*	Chang & Min, 2009[101]
	*RCAN1*	Chang & Min, 2009[101]
	*SYNJ1*	Voronov et al., 2008[29];
		Chang & Min, 2009[101]
Cancer and Leukemia	*ERG*	Ng et al., 2009[133]
	*ETS2*	Sussan et al., 2008[141]
	*RCAN1*	Baek et al., 2009[142]
	*RUNX1*	Edwards et al., 2009[136]
Heart Defects	Region between	Korbel et al., 2009[14]
	*DSCAM-ZNF295*	Korbel et al., 2009[14]

### Neurodevelopment

Neurodevelopment is known to be altered in people with DS. Already by mid-gestation the brains of fetuses with DS are smaller than those which do not have the condition. Cerebellar granule cells in Ts65Dn mice have reduced proliferation rates and elongation of the cell cycle length which could potentially result in a decrease in brain mass[51]; the number of these cells is also reduced in the Tc1 mouse model[33]. Neural progenitor cells (NPCs) from the Ts1Cje mouse model also exhibit similar defects as well as an increase in cell death[52]. The Ts1Cje and Ts2Cje mouse models have smaller brains, hypoplasia of the cerebellum, enlarged ventricles and decreased neurogenesis compared to euploid littermates[53]. The common region that is trisomic between these two mouse models contains approximately 86 genes (***Fig.1***), suggesting that this trisomic segment contains the causal dosage-sensitive genes for these detrimental developmental phenotypes[53],[54]. The decreased proliferation of cerebellar granule cells observed in the Ts65Dn mice has been attributed to a deficient mitotic response to the Sonic hedgehog (Shh) growth factor[55]. An altered response to Shh has also been demonstrated in Ts65Dn neural crest progenitor cells, and this may contribute to the craniofacial dysmorphology that is associated with DS[56],[57].

Elevated rates of neuronal apoptosis related to oxidative stress have been reported in DS[58]. Recent work suggests that Hsa21-encoded proteins PREP1, a transcription factor involved in the regulation of organism size[59], and tetratricopeptide repeat domain 3 (TTC3), an E3 ubiquitin ligase that targets AKT, a serine/threonine-protein kinase, may contribute to this phenotype[60]. Moreover, recent research provides evidence that oxidative stress is elevated in the Ts1Cje mouse, suggesting that one or more genes trisomic in this model, likely contribute to DS-associated oxidative stress[61]. Interestingly, aneuploidy of chromosomes other than Hsa21 also results in elevated apoptosis and reduced cellular proliferation[62],[63].

Recently, it was proposed that DYRK1A contributes to DS neural phenotypes, such as impaired dendritic growth, by disturbing neuron-restrictive silencer factor (REST/NRSF) levels[27],[64]. MicroRNAs encoded by Hsa21 may also influence development of the brain; specifically trisomy of miR-155 and miR-802 has been suggested to regulate the expression of the methyl-CpG-binding-protein gene (MECP2), which is known to be important in neurodevelopment[65].

### Pharmacological interventions to tackle brain and cognition in DS

This is a relatively new area of research in DS that is rapidly gaining momentum, and which arises from experiments carried out in mouse models in which behavioural, neurophysiological and cellular biology changes can be quantitatively assessed during development and ageing, and then modified through pharmacological intervention.

Several pharmacological interventions to enhance cognition in people with DS have been suggested, based upon efficacy in the Ts65Dn mouse (***Table 2***). Chronic treatment with gamma-aminobutyric acid (GABA) A receptor antagonists, picrotoxin or pentylenetetrazole, improved hippocampal-based learning and LTP deficits in Ts65Dn animals[40],[66],[67]. The GABA-ergic system regulates neuronal excitability throughout the nervous system and plays a significant role in cognition. Memantine, a non-competitive N-methyl-D-aspartic acid receptor (NMDAR) antagonist, has also been documented to improve learning in Ts65Dn mice[68], and is currently undergoing a clinical trial in a large group of DS patients[69]. Some clinical trials of Donepezil, an acetylcholinesterase inhibitor that is proposed to improve cholinergic neurotransmission, have reported small improvements in a subset of measures of cognition in people with DS[70]–[72]; however, not all Donepezil trials have demonstrated a statistically significant effect[73]–[76].

**Table 2:** Pharmacological interventions to tackle cognitive deficits in DS

Pharmacological Compound	Cognitive Effect	References
Donepezil	*Acetylcholinesterase inhibitor*	
	Limited success in DS patients	Spiridigliozzi et al., 2007[70]; Johnson et al., 2003[73]; Lott et al., 2002[74]; Prasher et al., 2002[75]
ECGC	*Natural polyphenol*	
	Attenuates cognitive deficits arising from DYRK1A overexpression	Guedj et al., 2009[79]
Fluoxetine	*Anti-depressant*	
	Prenatal treatment rescues impairments in neurogenesis	Clark et al., 2006[83]
L-DOPS or Xamoterol	*Norepinephrine prodrug β_1_ Adrenergic receptor partial antagonist*	
	Improves hippocampal-based contextual learning deficits in Ts65Dn	Salehi et al., 2009[77]
Lithium	*Mood stabilizer*	
	Prenatal treatment rescues impairments in neurogenesis	Bianchi et al., 2009[82]
Memantine	*Non-competitive NMDAR antagonist*	
	Improves learning in Ts65Dn	Costa et al., 2008[68]
	Currently undergoing clinical trial in DS patients	Mohan et al., 2009[69]
NAPVSIPQ & SALLRSIPA	*Neuroprotective peptides*	
	Prenatal treatment reverses developmental and glial deficits	Toso et al., 2008[84]
Picrotoxin or Pentlenetetrazole	*GABA(A) receptor antagonists*	
	Improves hippocampal-based learning and LTP deficits in Ts65Dn mouse model	Kleschevnikov et al., 2004[92];Fernandez et al., 2007[66];Reuda et al., 2008[67]
Vitamin E	*Antioxidant*	
	Partially rescues cognitive and morphological abnormalities in Ts65Dn	Lockrow et al., 2009[80]
	Reduces oxidation state of S100β	
		Bialowas-McGoey et al., 2008[81]

Recently, other pathways that modulate learning and memory have been examined with interest. Norepinephrine signaling in the hippocampus has been suggested to be impaired in the Ts65Dn mice because of degeneration of the locus coeruleus[77],[78]. In this model, learning deficits were reversed by treatment with a norepinephrine prodrug, L-DOPS, or xamoterol, a β1-adrenergic receptor partial antagonist. Interestingly, epigallocatechin gallate (ECGC), a natural polyphenol found in green tea leaves and is a specific inhibitor of DYRK1A, has been shown to attenuate cognitive defects arising from *DYRK1A* over-expression in transgenic mice[79]. Therapeutic interventions aimed at targeting oxidative imbalance report promising effects. Long-term supplementation with the antioxidant Vitamin E has been reported to partially rescue cognitive and morphological abnormalities in Ts65Dn mice[80], and reduce the oxidation state of S100 calcium binding protein beta (S100β), an Hsa21-encoded protein that is neurotoxic when in a reduced state[81].

Neurogenesis impairments in the Ts65Dn mice have been rescued by prenatal treatment with the mood-stabiliser, lithium, and by use of the anti-depressant, fluoxetine[82],[83]. Developmental delays and glial deficits in the Ts65Dn mouse model have been demonstrated to be partially reversed through prenatal treatment with neuroprotective peptides NAPVSIPQ+SALLRSIPA[84]. These results indicate that therapies during pregnancy could potentially improve developmental and glial deficits in DS.

The current findings are based on a thorough understanding of neuronal and cognitive deficits in mouse models of DS and are exciting in the therapeutic opportunities they offer. However, as with all pharmacological interventions, caution must be taken in translating findings from mice to humans.

### Alzheimer Disease in DS

A high incidence of early-onset Alzheimer Disease (AD) occurs in people with DS, with 30-70% of DS individuals developing dementia by the age of 60[4],[85]–[87]. AD pathology is characterized by brain atrophy, extracellular β-amyloid (Aβ) deposits and the accumulation of neurofibrillary tangles (NFTs) that are composed of hyperphosphorylated Tau. The amyloid precursor protein, amyloid precursor protein (APP), from which Aβ is produced, is encoded on Hsa21. In DS, the triplication of APP is proposed to be the underlying mechanism through which trisomy 21 individuals demonstrate an increased frequency of dementia[88]–[90].

Neurodegenerative phenotypes have also been observed in animal models of DS[77],[91]–[94]. In particular, loss of basal forebrain cholinergic neurons (BFCNs) occurs early in AD and is also observed in the Ts65Dn mouse[92],[93],[95]. Degeneration of these cells is related to a failure in the retrograde transport of nerve growth factor (NGF), and may arise from trisomy of *APP*[92]. Increased *APP* expression is also linked to enlargement of early endosomes[92],[95]–[99]. Recently, it was reported that lowering the expression of APP or beta-site APP-cleaving enzyme 1 (BACE-1), reversed endocytic abnormalities in fibroblasts derived from people with DS, and the over-expression of APP alone resulted in early endosome enlargements[100]. These data suggest that triplication of APP is sufficient to cause endosomal deficits, in contrast to previous reports[97]. Hsa21 genes other than APP may also contribute to endosomal phenotypes, in particular, overexpression of Hsa21 gene homologues in Drosophila, *dap160/ITSN1* (*intersectin1*)*, synj/SYNJ1* and *nla/RCAN1* (*runt-related tremscripthon factor 1*), results in abnormal synaptic morphology and impaired vesicle recycling[92],[101].

Other Hsa21 trisomic genes may also contribute to AD through different mechanisms. DYRK1A, an Hsa21 encoded kinase, phosphorylates Tau at a key priming site which may mediate its AD-related hyperphosphorylation in people with DS[102]. DYRK1A can also phosphorylate APP[103]. Indeed, increased phosphorylation of Tau has been reported in the Ts1Cje mouse model of DS that is not trisomic for APP[104] (***Fig. 1***). Mis-regulated splicing of Tau may contribute to NFT formation in AD[105],[106]. PCBP3, an Hsa21 protein, modifies splicing of Tau and may contribute to the expression of AD associated Tau isoforms in people with DS[107]. Recently, degeneration of Purkinje cells in the cerebellum of aged Ts65Dn mice, proximal to deposits of Aβ and Tau, has been observed[94],[108].

### Other neurological disorders

Six percent of children and adolescents with DS have epileptic seizures[109]. Children with DS are also susceptible to infantile spasms, however little is known about the molecular mechanisms underlying this. Treating Ts65Dn mice with GABA(B) receptor agonists induced a phenotype reminiscent of infantile spasms, providing a model to further understand the pathogenesis of this phenotype[110]. Moyamoya syndrome, a cerebrovascular condition that is characterized by reduced blood flow predisposing to stroke[111], has been reported to occur with a higher frequency in people with DS than in the general population[112]. Recently, the expression of β-catenin was found to be increased in brain capillary endothelial cells in the Ts65Dn mouse model, however whether this finding is linked to Moyamoya syndrome is as yet unclear[113],[114].

People with DS have been reported to experience disturbed sleeping patterns. Studies of circadian activity in the Ts65Dn mouse model have reported conflicting results of both intact[25],[115],[116] and disturbed rhythms[117]. Future studies of this phenotype in alternative mouse models of DS will thus be of value.

### Cancer and leukemia

Children with DS have a greatly elevated risk of developing the otherwise very rare transient myeloproliferative disorder (TMD), as well as acute megakaryocytic leukemia (AMKL) and acute lymphoblastic leukemia (ALL)[6],[118],[119]. Trisomy of Hsa21 leads to an expansion of the megakaryocyte-erythroid progenitor population[120],[121], which precedes the development of TMD. The development of TMD and AMKL is almost always associated with stereotypical mutations in exon 2 of the *GATA binding protein 1* (*GATA1*) gene resulting in the synthesis of a truncated GATA1 protein termed GATA1s[6],[122],[123]. Mutations in Janus kinase 3 (JAK3) have also been reported by several groups to be associated with AMKL[119],[124]–[128]. Additionally, one fifth of DS-ALL cases have been associated with janus kinase 2 (JAK2) point mutations[129],[130]. DS-ALL is also associated with aberrant expression of cyto kine receptor-like factor 2 (CRLF2) linked to genomic rearrangements[130]–[132]. Trisomy of an Hsa21-encoded gene, *v-ets erythroblastosis virus E26 oncogene homolog* (*ERG*), is required for development of the myeloproliferation defect in the Ts65Dn model[133]. The Hsa21 gene *runt-related transcription factor 1* (*RUNX1*) has also been proposed to regulate hematopoiesis via the phosphoinositide 3 (PI3)-kinase/AKT pathway[134]–[136].

Despite perturbations of hematopoietic development in the Ts1Cje, Ts65Dn and Tc1 models of DS, these mice do not develop leukaemia, even when the trisomic models also express disease-associated GATA1 mutations[137]–[139]. It is possible that trisomy of Hsa21 genes other than those encoded in these models, in concert with mutations in non-Hsa21 encoded genes such as GATA1, JAK3 or CRLF2, may be required for the development of leukemia.

Although DS is associated with a predisposition to leukemia, people with DS have a reduced risk of developing most solid tumours[7],[140]. Crossing a mouse model of colon cancer, Apc^min^, with mouse models of DS resulted in reduced formation of tumors, dependent on the trisomy of the Hsa21-encoded *ETS2* gene[141]. Recently overexpression of the Hsa21 gene, *regulator of calcineurin* (*RCAN1*), was shown to be sufficient to suppress tumour growth by attenuating angiogenesis via the regulation of vascular endothelial growth factor (VEGF) signaling[142]. However, in a Ts65Dn trisomic background removal of one copy of *Rcan1* did not completely abrogate the effect of trisomy on tumour formation, suggesting that other Hsa21 genes also contribute to this phenotype[142].

### Heart defects

Congenital heart defects (CHD) are prevalent in 40% of children with DS and over 50% of all atrioventricular septal heart defects (AVSDs) in infancy are attributed to trisomy Hsa21[5],[143]. Mutations in *cysteine*-*rich with EGF*-*like domains 1*(*CRELD1*), a non-Hsa21 gene, contribute to the occurrence of AVSD in DS[144]. Several DS mouse models exhibit heart defects reminiscent of those in DS[18],[33],[63],[145], suggesting that trisomic genes common to these models influence the development of the heart. Analysis of the occurrence of CHD in people who have partial trisomies of Hsa21 has suggested that trisomy of genes within a 1.77 Mb region [*DSCAM- ZNF295* (*zinc finger protein 295*)] of Hsa21 may be sufficient for the development of CHD[14].

## CONCLUSION

DS is complex disorder and dissecting the genetic and molecular processes underlying the syndrome requires many different complementary approaches, including the study of human data and mouse and other model organisms. However, several recent breakthroughs have increased our understanding of the effects of Hsa21 trisomy. Combining information from studies of people with DS with the power of mouse models of trisomy has enabled genetic associations to be tested and continues to lead to the identification of genes that cause DS-associated pathology. Significant advances in basic research have been instrumental in determining the molecular mechanisms underlying these phenotypes leading to useful therapeutic interventions. However, many aspects of DS crucial to the health and well-being of people with the condition remain to be investigated and require study at all levels.

## References

[1] Gardiner K (2010). J. Molecular basis of pharmacotherapies for cognition in Down syndrome. Trends Pharmacol Sci.

[2] Hassold T, Abruzzo M, Adkins K, Griffin D, Merrill M, Millie E (1996). Human aneuploidy: incidence, origin, and etiology. Environ Mol Mutagen.

[3] Antonarakis SE, Lyle R, Dermitzakis ET, Reymond A (2004). Deutsch S. Chromosome 21 and down syndrome: from genomics to pathophysiology. Nat Rev Genet.

[4] Johannsen P, Christensen JE, Mai J (1996). The prevalence of dementia in Down syndrome. Dementia.

[5] Freeman SB, Bean LH, Allen EG, Tinker SW, Locke AE, Druschel C (2008). Ethnicity, sex, and the incidence of congenital heart defects: a report from the National Down Syndrome Project. Genet Med.

[6] Wechsler J, Greene M, McDevitt MA, Anastasi J, Karp JE, Le Beau MM (2002). Acquired mutations in GATA1 in the megakaryoblastic leukemia of Down syndrome. Nat Genet.

[7] Hasle H (2001). Pattern of malignant disorders in individuals with Down's syndrome. Lancet Oncol.

[8] Bittles AH, Glasson EJ (2004). Clinical, social, and ethical implications of changing life expectancy in Down syndrome. Dev Med Child Neurol.

[9] Prandini P, Deutsch S, Lyle R, Gagnebin M, Delucinge VC, Delorenzi M (2007). Natural gene-expression variation in Down syndrome modulates the outcome of gene-dosage imbalance. Am J Hum Genet.

[10] Ait Yahya-Graison E, Aubert J, Dauphinot L, Rivals I, Prieur M, Golfier G (2007). Classification of human chromosome 21 gene-expression variations in Down syndrome: impact on disease phenotypes. Am J Hum Genet.

[11] Sultan M, Piccini I, Balzereit D, Herwig R, Saran NG, Lehrach H (2007). Gene expression variation in Down's syndrome mice allows prioritization of candidate genes. Genome Biol.

[12] Pritchard MA, Kola I (1999). The “gene dosage effect” hypothesis versus the “amplified developmental instability” hypothesis in Down syndrome. J Neural Transm Suppl.

[13] Olson LE, Roper RJ, Sengstaken CL, Peterson EA, Aquino V, Galdzicki Z (2007). Trisomy for the Down syndrome ‘critical region’ is necessary but not sufficient for brain phenotypes of trisomic mice. Hum Mol Genet.

[14] Korbel JO, Tirosh-Wagner T, Urban AE, Chen XN, Kasowski M, Dai L (2009). The genetic architecture of Down syndrome phenotypes revealed by high-resolution analysis of human segmental trisomies. PNAS.

[15] Lyle R, Bena F, Gagos S, Gehrig C, Lopez G, Schinzel A (2009). Genotype-phenotype correlations in Down syndrome identified by array CGH in 30 cases of partial trisomy and partial monosomy chromosome 21. Eur J Hum Genet.

[16] Brault V, Besson V, Magnol L, Duchon A, Herault Y (2007). Cre/loxP-mediated chromosome engineering of the mouse genome. Handb Exp Pharmacol.

[17] Duchon A, Besson V, Pereira PL, Magnol L, Herault Y (2008). Inducing segmental aneuploid mosaicism in the mouse through targeted asymmetric sister chromatid event of recombination. Genetics.

[18] Li Z, Yu T, Morishima M, Pao A, LaDuca J, Conroy J (2007). Duplication of the entire 22.9 Mb human chromosome 21 syntenic region on mouse chromosome 16 causes cardiovascular and gastrointestinal abnormalities. Hum Mol Genet.

[19] Olson LE, Richtsmeier JT, Leszl J, Reeves RH (2004). A chromosome 21 critical region does not cause specific Down syndrome phenotypes. Science.

[20] Brault V, Pereira P, Duchon A, Herault Y (2006). Modeling chromosomes in mouse to explore the function of genes, genomic disorders, and chromosomal organization. PLoS Genet.

[21] Besson V, Brault V, Duchon A, Togbe D, Bizot JC, Quesniaux VF (2007). Modeling the monosomy for the telomeric part of human chromosome 21 reveals haploinsufficient genes modulating the inflammatory and airway responses. Hum Mol Genet.

[22] Pereira PL, Magnol L, Sahun I, Brault V, Duchon A, Prandini P (2009). A new mouse model for the trisomy of the Abcg1-U2af1 region reveals the complexity of the combinatorial genetic code of down syndrome. Hum Mol Genet.

[23] Villar AJ, Belichenko PV, Gillespie AM, Kozy HM, Mobley WC, Epstein CJ (2005). Identification and characterization of a new Down syndrome model, Ts[Rb(12.1716)]2Cje, resulting from a spontaneous Robertsonian fusion between T(171)65Dn and mouse chromosome 12. Mamm Genome.

[24] Sago H, Carlson EJ, Smith DJ, Kilbridge J, Rubin EM, Mobley WC (1998). Ts1Cje, a partial trisomy 16 mouse model for Down syndrome, exhibits learning and behavioral abnormalities. PNAS.

[25] Reeves RH, Irving NG, Moran TH, Wohn A, Kitt C, Sisodia SS (1995). A mouse model for Down syndrome exhibits learning and behaviour deficits. Nat Genet.

[26] Ahn KJ, Jeong HK, Choi HS, Ryoo SR, Kim YJ, Goo JS (2006). DYRK1A BAC transgenic mice show altered synaptic plasticity with learning and memory defects. Neurobiol Dis.

[27] Lepagnol-Bestel AM, Zvara A, Maussion G, Quignon F, Ngimbous B, Ramoz N (2009). DYRK1A interacts with the REST/NRSF-SWI/SNF chromatin remodelling complex to deregulate gene clusters involved in the neuronal phenotypic traits of Down syndrome. Hum Mol Genet.

[28] Best TK, Siarey R J, Galdzicki Z (2007). Ts65Dn, a mouse model of Down syndrome, exhibits increased GABAB-induced potassium current. J Neurophysiol.

[29] Voronov SV, Frere SG, Giovedi S, Pollina EA, Borel C, Zhang H (2008). Synaptojanin 1-linked phosphoinositide dyshomeostasis and cognitive deficits in mouse models of Down's syndrome. PNAS.

[30] Altafaj X, Dierssen M, Baamonde C, Marti E, Visa J, Guimera J (2001). Neurodevelopmental delay, motor abnormalities and cognitive deficits in transgenic mice overexpressing Dyrk1A (minibrain), a murine model of Down's syndrome. Hum Mol Genet.

[31] Chrast R, Scott HS, Madani R, Huber L, Wolfer DP, Prinz M (2000). Mice trisomic for a bacterial artificial chromosome with the single-minded 2 gene (Sim2) show phenotypes similar to some of those present in the partial trisomy 16 mouse models of Down syndrome. Hum Mol Genet.

[32] Ema M, Ikegami S, Hosoya T, Mimura J, Ohtani H, Nakao K (1999). Mild impairment of learning and memory in mice overexpressing the mSim2 gene located on chromosome 16: an animal model of Down's syndrome. Hum Mol Genet.

[33] O'Doherty A, Ruf S, Mulligan C, Hildreth V, Errington ML, Cooke S (2005). An aneuploid mouse strain carrying human chromosome 21 with Down syndrome phenotypes. Science.

[34] Vicari S, Carlesimo GA (2006). Short-term memory deficits are not uniform in Down and Williams syndromes. Neuropsychol Rev.

[35] Carlesimo GA, Marotta L, Vicari S (1997). Long-term memory in mental retardation: evidence for a specific impairment in subjects with Down's syndrome. Neuropsychologia.

[36] Weis S, Weber G, Neuhold A, Rett A (1991). Down syndrome: MR quantification of brain structures and comparison with normal control subjects. AJNR.

[37] Aylward EH, Habbak R, Warren AC, Pulsifer MB, Barta PE, Jerram M (1997). Cerebellar volume in adults with Down syndrome. Arch Neurol.

[38] Pearlson GD, Breiter SN, Aylward EH, Warren AC, Grygorcewicz M, Frangou S (1998). MRI brain changes in subjects with Down syndrome with and without dementia. Dev Med Child Neurol.

[39] Aylward EH, Li Q, Honeycutt NA, Warren AC, Pulsifer MB, Barta P E (1999). MRI volumes of the hippocampus and amygdala in adults with Down's syndrome with and without dementia. Am J Psychiatry.

[40] Kleschevnikov AM, Belichenko PV, Villar AJ, Epstein CJ, Malenka RC, Mobley WC (2004). Hippocampal long-term potentiation suppressed by increased inhibition in the Ts65Dn mouse, a genetic model of Down syndrome. J Neurosci.

[41] Belichenko PV, Masliah E, Kleschevnikov AM, Villar AJ, Epstein CJ, Salehi A (2004). Synaptic structural abnormalities in the Ts65Dn mouse model of Down Syndrome. J Comp Neurol.

[42] Belichenko PV, Kleschevnikov AM, Masliah E, Wu C, Takimoto-Kimura R, Salehi A (2009). Excitatory-inhibitory relationship in the fascia dentata in the Ts65Dn mouse model of Down syndrome. J Comp Neurol.

[43] Di Filippo M, Tozzi A, Ghiglieri V, Picconi B, Costa C, Cipriani S (2009). Impaired plasticity at specific subset of striatal synapses in the Ts65Dn mouse model of Down syndrome. Biol Psychiatry.

[44] Morice E, Andreae LC, Cooke SF, Vanes L, Fisher EM, Tybulewicz VL (2008). Preservation of long-term memory and synaptic plasticity despite short-term impairments in the Tc1 mouse model of Down syndrome. Learn Mem.

[45] Galante M, Jani H, Vanes L, Daniel H, Fisher EM, Tybulewicz VL (2009). Impairments in motor coordination without major changes in cerebellar plasticity in the Tc1 mouse model of Down syndrome. Hum Mol Genet.

[46] Belichenko NP, Belichenko PV, Kleschevnikov AM, Salehi A, Reeves RH, Mobley WC (2000). The “Down syndrome critical region” is sufficient in the mouse model to confer behavioral, neurophysiological, and synaptic phenotypes characteristic of Down syndrome. J Neurosci.

[47] Best TK, Cho-Clark M, Siarey RJ, Galdzicki Z (2008). Speeding of miniature excitatory post-synaptic currents in Ts65Dn cultured hippocampal neurons. Neurosci Lett.

[48] Meng X, Shi J, Peng B, Zou X, Zhang C (2006). Effect of mouse Sim2 gene on the cell cycle of PC12 cells. Cell Biol Int.

[49] Rachidi M, Delezoide AL, Delabar JM, Lopes C (2009). A quantitative assessment of gene expression (QAGE) reveals differential overexpression of DOPEY2, a candidate gene for mental retardation, in Down syndrome brain regions. Int J Dev Neurosci.

[50] Yu HH, Yang JS, Wang J, Huang Y, Lee T (2009). Endodomain diversity in the Drosophila Dscam and its roles in neuronal morphogenesis. J Neurosci.

[51] Contestabile A, Fila T, Bartesaghi R, Ciani E (2009). Cell cycle elongation impairs proliferation of cerebellar granule cell precursors in the Ts65Dn mouse, an animal model for Down syndrome. Brain Pathol.

[52] Moldrich RX, Dauphinot L, Laffaire J, Vitalis T, Herault Y, Beart PM (2009). Proliferation deficits and gene expression dysregulation in Down's syndrome (Ts1Cje) neural progenitor cells cultured from neurospheres. J Neurosci Res.

[53] Ishihara K, Amano K, Takaki E, Shimohata A, Sago H, Epstein J (2009). Enlarged brain ventricles and impaired neurogenesis in the Ts1Cje and Ts2Cje mouse models of Down syndrome. Cereb Cortex.

[54] Laffaire J, Rivals I, Dauphinot L, Pasteau F, Wehrle R, Larrat B (2009). Gene expression signature of cerebellar hypoplasia in a mouse model of Down syndrome during postnatal development. BMC Genomics.

[55] Roper RJ, Baxter LL, Saran NG, Klinedinst DK, Beachy PA, Reeves RH (2006). Defective cerebellar response to mitogenic Hedgehog signaling in Down [corrected] syndrome mice. PNAS.

[56] Roper RJ, VanHorn JF, Cain CC, Reeves RH (2009). A neural crest deficit in Down syndrome mice is associated with deficient mitotic response to Sonic hedgehog. Mech Dev.

[57] Richtsmeier JT, Baxter LL, Reeves RH (2000). Parallels of craniofacial maldevelopment in Down syndrome and Ts65Dn mice. Dev Dyn.

[58] Busciglio J, Yankner BA (1995). Apoptosis and increased generation of reactive oxygen species in Down's syndrome neurons in vitro. Nature.

[59] Micali N, Longobardi E, Iotti G, Ferrai C, Castagnaro L, Ricciardi M (2010). Down syndrome fibroblasts and mouse Prep1-overexpressing cells display increased sensitivity to genotoxic stress. Nucleic Acids Res.

[60] Suizu F, Hiramuki Y, Okumura F, Matsuda M, Okumura AJ, Hirata N (2009). The E3 ligase TTC3 facilitates ubiquitination and degradation of phosphorylated Akt. Dev Cell.

[61] Ishihara K, Amano K, Takaki E, Ebrahim AS, Shimohata A, Shibazaki N (2009). Increased lipid peroxidation in Down's syndrome mouse models. J Neurochem.

[62] Kai Y, Wang CC, Kishigami S, Kazuki Y, Abe S, Takiguchi M (2009). Enhanced apoptosis during early neuronal differentiation in mouse ES cells with autosomal imbalance. Cell Res.

[63] Williams AD, Mjaatvedt CH, Moore CS (2008). Characterization of the cardiac phenotype in neonatal Ts65Dn mice. Dev Dyn.

[64] Canzonetta C, Mulligan C, Deutsch S, Ruf S, O'Doherty A, Lyle R (2008). DYRK1A-dosage imbalance perturbs NRSF/REST levels, deregulating pluripotency and embryonic stem cell fate in Down syndrome. Am J Hum Genet.

[65] Kuhn DE, Nuovo GJ, Terry AV, Martin MM, Malana GE, Sansom SE (2010). Chromosome 21-derived microRNAs provide an etiological basis for aberrant protein expression in human Down syndrome brains. J Biol Chem.

[66] Fernandez F, Morishita W, Zuniga E, Nguyen J, Blank M, Malenka RC (2007). Pharmacotherapy for cognitive impairment in a mouse model of Down syndrome. Nat Neurosci.

[67] Rueda N, Florez J, Martinez-Cue C (2008). Chronic pentylenetetrazole but not donepezil treatment rescues spatial cognition in Ts65Dn mice, a model for Down syndrome. Neurosci Lett.

[68] Costa AC, Scott-McKean JJ, Stasko MR (2008). Acute injections of the NMDA receptor antagonist memantine rescue performance deficits of the Ts65Dn mouse model of Down syndrome on a fear conditioning test. Neuropsychopharmacology.

[69] Mohan M, Bennett C, Carpenter PK (2009). Memantine for dementia in people with Down syndrome. Cochrane. Database.Syst.Rev.

[70] Spiridigliozzi GA, Heller JH, Crissman BG, Sullivan-Saarela JA, Eells R, Dawson D (2007). Preliminary study of the safety and efficacy of donepezil hydrochloride in children with Down syndrome: a clinical report series. Am J Med Genet A.

[71] Heller JH, Spiridigliozzi GA, Sullivan JA, Doraiswamy PM, Krishnan RR, Kishnani PS (2003). Donepezil for the treatment of language deficits in adults with Down syndrome: a preliminary 24-week open trial. Am J Med Genet A.

[72] Heller JH, Spiridigliozzi GA, Doraiswamy PM, Sullivan JA, Crissman BG, Kishnani PS (2004). Donepezil effects on language in children with Down syndrome: results of the first 22-week pilot clinical trial. Am J Med Genet A.

[73] Johnson N, Fahey C, Chicoine B, Chong G, Gitelman D (2003). Effects of donepezil on cognitive functioning in Down syndrome. Am J Ment Retard.

[74] Lott IT, Osann K, Doran E, Nelson L (2002). Down syndrome and Alzheimer disease: response to donepezil. Arch Neurol.

[75] Prasher VP, Huxley A, Haque MS (2002). A 24-week, double-blind, placebo-controlled trial of donepezil in patients with Down syndrome and Alzheimer's disease--pilot study. Int J Geriatr Psychiatr.

[76] Kishnani PS, Sommer BR, Handen BL, Seltzer B, Capone GT, Spiridigliozzi GA (2009). The efficacy, safety, and tolerability of donepezil for the treatment of young adults with Down syndrome. Am J Med Genet A.

[77] Salehi A, Faizi M, Colas D, Valletta J, Laguna J, Takimoto-Kimura R (2009). Restoration of norepinephrine-modulated contextual memory in a mouse model of Down syndrome. Science Translational Medicine.

[78] Wiseman FK (2009). Cognitive enhancement therapy for a model of Down syndrome. Science Translational Medicine.

[79] Guedj F, Sebrie C, Rivals I, Ledru A, Paly E, Bizot JC (2009). Green tea polyphenols rescue of brain defects induced by overexpression of DYRK1A. PLoS.One.

[80] Lockrow J, Prakasam A, Huang P, Bimonte-Nelson H, Sambamurti K, Granholm AC (2009). Cholinergic degeneration and memory loss delayed by vitamin E in a Down syndrome mouse model. Exp Neurol.

[81] Bialowas-McGoey LA, Lesicka A, Whitaker-Azmitia PM (2008). Vitamin E increases S100B-mediated microglial activation in an S100B-overexpressing mouse model of pathological aging. Glia.

[82] Bianchi P, Ciani E, Contestabile A, Guidi S, Bartesaghi R (2009). Lithium restores neurogenesis in the subventricular zone of the Ts65Dn mouse, a model for Down syndrome. Brain Pathol DOI.

[83] Clark S, Schwalbe J, Stasko MR, Yarowsky PJ, Costa AC (2006). Fluoxetine rescues deficient neurogenesis in hippocampus of the Ts65Dn mouse model for Down syndrome. Exp Neurol.

[84] Toso L, Cameroni I, Roberson R, Abebe D, Bissell S, Spong CY (2008). Prevention of developmental delays in a Down syndrome mouse model. Obstet. Gynecol.

[85] Holland AJ, Hon J, Huppert FA, Stevens F (2000). Incidence and course of dementia in people with Down's syndrome: findings from a population-based study. J Intellect Disabil Res.

[86] Holland AJ, Hon J, Huppert FA, Stevens F, Watson P (1998). Population-based study of the prevalence and presentation of dementia in adults with Down's syndrome. Br J Psychiatry.

[87] Coppus A, Evenhuis H, Verberne GJ, Visser F, van Gool P, Eikelenboom P (2006). Dementia and mortality in persons with Down's syndrome. J Intellect Disabil Res.

[88] Rovelet-Lecrux A, Hannequin D, Raux G, Le Meur N, Laquerriere A, Vital A (2006). APP locus duplication causes autosomal dominant early-onset Alzheimer disease with cerebral amyloid angiopathy. Nat Genet.

[89] Sleegers K, Brouwers N, Gijselinck I, Theuns J, Goossens D, Wauters J (2006). APP duplication is sufficient to cause early onset Alzheimer's dementia with cerebral amyloid angiopathy. Brain.

[90] Cabrejo L, Guyant-Marechal L, Laquerriere A, Vercelletto M, De la Fourniere F, Thomas-Anterion C (2006). Phenotype associated with APP duplication in five families. Brain.

[91] Hunter CL, Bimonte HA, Granholm AC (2003). Behavioral comparison of 4 and 6 month-old Ts65Dn mice: age-related impairments in working and reference memory. Behav. Brain Res.

[92] Salehi A, Delcroix JD, Belichenko PV, Zhan K, Wu C, Va;;etta JS (2006). Increased App expression in a mouse model of Down's syndrome disrupts NGF transport and causes cholinergic neuron degeneration. Neuron.

[93] Granholm AC, Sanders LA, Crnic LS (2000). Loss of cholinergic phenotype in basal forebrain coincides with cognitive decline in a mouse model of Down's syndrome. Exp Neurol.

[94] Necchi D, Lomoio S, Scherini E (2008). Axonal abnormalities in cerebellar Purkinje cells of the Ts65Dn mouse. Brain Res.

[95] Seo H, Isacson O (2005). Abnormal APP, cholinergic and cognitive function in Ts65Dn Down's model mice. Exp Neurol.

[96] Cataldo AM, Barnett JL, Pieroni C, Nixon RA (1997). Increased neuronal endocytosis and protease delivery to early endosomes in sporadic Alzheimer's disease: neuropathologic evidence for a mechanism of increased beta-amyloidogenesis. J Neurosci.

[97] Cataldo AM, Petanceska S, Peterhoff CM, Terio NB, Epstein CJ, Villar A (2003). App gene dosage modulates endosomal abnormalities of Alzheimer's disease in a segmental trisomy 16 mouse model of Down syndrome. J Neurosci.

[98] Cataldo AM, Mathews PM, Boiteau AB, Hassinger LC, Peterhoff CM, Jiang Y (2008). Down syndrome fibroblast model of Alzheimer-related endosome pathology: accelerated endocytosis promotes late endocytic defects. Am J Pathol.

[99] Cooper JD, Salehi A, Delcroix JD, Howe CL, Belichenko PV, Chua-Couzens J (2001). Failed retrograde transport of NGF in a mouse model of Down's syndrome: reversal of cholinergic neurodegenerative phenotypes following NGF infusion. PNAS.

[100] Jiang Y, Mullaney KA, Peterhoff CM, Che S, Schmidt SD, Boyer-Boiteau A (2010). Alzheimer's-related endosome dysfunction in Down syndrome is Abeta-independent but requires APP and is reversed by BACE-1 inhibition. PNAS.

[101] Chang KT, Min KT (2009). Upregulation of three Drosophila homologs of human chromosome 21 genes alters synaptic function: implications for Down syndrome. PNAS.

[102] Ryoo SR, Jeong HK, Radnaabazar C, Yoo JJ, Cho HJ, Lee HW (2007). DYRK1A-mediated hyperphosphorylation of Tau. A functional link between Down syndrome and Alzheimer disease. J Biol Chem.

[103] Ryoo SR, Cho HJ, Lee HW, Jeong HK, Radnaabazar C, Kim YS (2008). Dual-specificity tyrosine (Y)-phosphorylation regulated kinase 1A-mediated phosphorylation of amyloid precursor protein: evidence for a functional link between Down syndrome and Alzheimer's disease. J Neurochem.

[104] Shukkur EA, Shimohata A, Akagi T, Yu W, Yamaguchi M, Murayama M (2006). Mitochondrial dysfunction and tau hyperphosphorylation in Ts1Cje, a mouse model for Down syndrome. Hum Mol Genet.

[105] Liu F, Liang Z, Wegiel J, Hwang YW, Iqbal K, Grundke-Iqbal I (2008). Overexpression of Dyrk1A contributes to neurofibrillary degeneration in Down syndrome. FASEB J.

[106] Woods YL, Cohen P, Becker W, Jakes R, Goedert M, Wang X (2001). The kinase DYRK phosphorylates protein-synthesis initiation factor eIF2Bepsilon at Ser539 and the microtubule-associated protein tau at Thr212: potential role for DYRK as a glycogen synthase kinase 3-priming kinase. Biochem J.

[107] Wang Y, Gao L, Tse SW, Andreadis A (2010). Heterogeneous nuclear ribonucleoprotein E3 modestly activates splicing of tau exon 10 via its proximal downstream intron, a hotspot for frontotemporal dementia mutations. Gene.

[108] Lomoio S, Scherini E, Necchi D (2009). Beta-amyloid overload does not directly correlate with SAPK/JNK activation and tau protein phosphorylation in the cerebellar cortex of Ts65Dn mice. Brain Res.

[109] Smigielska-Kuzia J, Sobaniec W, Kulak W, Bockowski L (2009). Clinical and EEG features of epilepsy in children and adolescents in Down syndrome. J Child Neurol.

[110] Cortez MA, Shen L, Wu Y, Aleem IS, Trepanier CH, Sadeghnia HR (2009). Infantile spasms and Down syndrome: a new animal model. Pediatr Res.

[111] Scott RM, Smith ER (2009). Moyamoya disease and moyamoya syndrome. N Engl J Med.

[112] Fukushima Y, Kondo Y, Kuroki Y, Miyake S, Iwamoto H, Sekido K (1986). Are Down syndrome patients predisposed to Moyamoya disease?. Eur J Pediatr.

[113] Ramakrishna N, Meeker HC, Li S, Brown WT, Rao R, El Idrissi A (2009). Upregulation of beta-catenin expression in Down syndrome model Ts65Dn mouse brain. Neuroscience.

[114] Vorbrodt AW, Li S, Brown WT, Ramakrishna N (2008). Increased expression of beta-catenin in brain microvessels of a segmentally trisomic (Ts65Dn) mouse model of Down syndrome. Brain Cell Biol.

[115] Ruby NF, Fernandez F, Zhang P, Klima J, Heller HC, Garner CC (2010). Circadian locomotor rhythms are normal in Ts65Dn “down syndrome” mice and unaffected by pentylenetetrazole. J Biol Rhythms.

[116] Martinez-Cue C, Baamonde C, Lumbreras M, Paz J, Davisson MT, Schmidt C (2002). Differential effects of environmental enrichment on behavior and learning of male and female Ts65Dn mice, a model for Down syndrome. Behav Brain Res.

[117] Stewart RE, Woodhouse JM, Cregg M, Pakeman VH (2007). Association between accommodative accuracy, hypermetropia, and strabismus in children with Down's syndrome. Optom Vis Sci.

[118] Izraeli S, Rainis L, Hertzberg L, Smooha G, Birger Y (2007). Trisomy of chromosome 21 in leukemogenesis. Blood Cells Mol Dis.

[119] Malinge S, Ben Abdelali R, Settegrana C, Radford-Weiss I, Debre M, Beldjord K (2007). Novel activating JAK2 mutation in a patient with Down syndrome and B-cell precursor acute lymphoblastic leukemia. Blood.

[120] Chou ST, Opalinska JB, Yao Y, Fernandes MA, Kalota A, Brooks JS (2008). Trisomy 21 enhances human fetal erythro-megakaryocytic development. Blood.

[121] Tunstall-Pedoe O, Roy A, Karadimitris A, de la Fuente J, Fisk NM, Bennett P (2008). Abnormalities in the myeloid progenitor compartment in Down syndrome fetal liver precede acquisition of GATA1 mutations. Blood.

[122] Groet J, McElwaine S, Spinelli M, Rinaldi A, Burtscher I, Mulligan C (2003). Acquired mutations in GATA1 in neonates with Down's syndrome with transient myeloid disorder. Lancet.

[123] Stepensky P, Brooks R, Waldman E, Revel-Vilk S, Izraeli S, Resnick I (2010). A rare case of GATA1 negative chemoresistant acute megakaryocytic leukemia in an 8-month-old infant with trisomy 21. Pediatr Blood Cancer.

[124] Sato T, Toki T, Kanezaki R, Xu G, Terui K, Kanegane H (2008). Functional analysis of JAK3 mutations in transient myeloproliferative disorder and acute megakaryoblastic leukaemia accompanying Down syndrome. Br J Haematol.

[125] Klusmann JH, Reinhardt D, Hasle H, Kaspers GJ, Creutzig U, Hahlen K (2007). Janus kinase mutations in the development of acute megakaryoblastic leukemia in children with and without Down's syndrome. Leukemia.

[126] Kiyoi H, Yamaji S, Kojima S, Naoe T (2007). JAK3 mutations occur in acute megakaryoblastic leukemia both in Down syndrome children and non-Down syndrome adults. Leukemia.

[127] Walters DK, Mercher T, Gu TL, O'Hare T, Tyner JW, Loriaux M (2006). Activating alleles of JAK3 in acute megakaryoblastic leukemia. Cancer Cell.

[128] De Vita S, Mulligan C, McElwaine S, Dagna-Bricarelli F, Spinelli M, Basso G (2007). Loss-of-function JAK3 mutations in TMD and AMKL of Down syndrome. Br J Haematol.

[129] Gaikwad A, Rye CL, Devidas M, Heerema NA, Carroll AJ, Izraeli S (2009). Prevalence and clinical correlates of JAK2 mutations in Down syndrome acute lymphoblastic leukaemia. Br J Haematol.

[130] Hertzberg L, Vendramini E, Ganmore I, Cazzaniga G, Schmitz M, Chalker J (2010). Down syndrome acute lymphoblastic leukemia, a highly heterogeneous disease in which aberrant expression of CRLF2 is associated with mutated JAK2: a report from the International BFM Study Group. Blood.

[131] Russell LJ, Capasso M, Vater I, Akasaka T, Bernard OA, Calasanz MJ (2009). Deregulated expression of cytokine receptor gene, CRLF2, is involved in lymphoid transformation in B-cell precursor acute lymphoblastic leukemia. Blood.

[132] Mullighan CG, Collins-Underwood JR, Phillips LA, Loudin MG, Liu W, Zhang J (2009). Rearrangement of CRLF2 in B-progenitor- and Down syndrome-associated acute lymphoblastic leukemia. Nat Genet.

[133] Ng AP, Hyland CD, Metcalf D, Carmichael CL, Loughran SJ, Di Rago L (2009). Trisomy of Erg is required for myeloproliferation in a mouse model of Down syndrome. Blood DOI.

[134] Lutterbach B, Hiebert SW (2000). Role of the transcription factor AML-1 in acute leukemia and hematopoietic differentiation. Gene.

[135] Okuda T, van Deursen J, Hiebert SW, Grosveld G, Downing JR (1996). AML1, the target of multiple chromosomal translocations in human leukemia, is essential for normal fetal liver hematopoiesis. Cell.

[136] Edwards H, Xie C, LaFiura KM, Dombkowski AA, Buck SA, Boerner JL (2009). RUNX1 regulates phosphoinositide 3-kinase/AKT pathway: role in chemotherapy sensitivity in acute megakaryocytic leukemia. Blood.

[137] Carmichael CL, Majewski IJ, Alexander WS, Metcalf D, Hilton DJ, Hewitt CA (2009). Hematopoietic defects in the Ts1Cje mouse model of Down syndrome. Blood.

[138] Alford K, Slender A, Vanes L, Li Z, Fisher EM, NizeticD, et al Perturbed hematopoiesis in the Tc1 mouse model of Down Syndrome. Blood.

[139] Kirsammer G, Jilani S, Liu H, Davis E, Gurbuxani S, Le Beau MM (2008). Highly penetrant myeloproliferative disease in the Ts65Dn mouse model of Down syndrome. Blood.

[140] Yang Q, Rasmussen SA, Friedman J M (2002). Mortality associated with Down's syndrome in the USA from 1983 to 1997: a population-based study. Lancet.

[141] Sussan TE, Yang A, Li F, Ostrowski MC, Reeves RH (2008). Trisomy represses Apc(Min)-mediated tumours in mouse models of Down's syndrome. Nature.

[142] Baek KH, Zaslavsky A, Lynch RC, Britt C, Okada Y, Siarey RJ (2009). Down's syndrome suppression of tumour growth and the role of the calcineurin inhibitor DSCR1. Nature.

[143] Delom F, Burt E, Hoischen A, Veltman J, Groet J, Cotter FE (2009). Transchromosomic cell model of Down syndrome shows aberrant migration, adhesion and proteome response to extracellular matrix. Proteome Sci.

[144] Maslen CL, Babcock D, Robinson SW, Bean LJ, Dooley KJ, Willour VL (2006). CRELD1 mutations contribute to the occurrence of cardiac atrioventricular septal defects in Down syndrome. Am J Med Genet A.

[145] Moore CS (2006). Postnatal lethality and cardiac anomalies in the Ts65Dn Down syndrome mouse model. Mamm. Genome.

